# Two-month stop in mammographic screening significantly impacts on breast cancer stage at diagnosis and upfront treatment in the COVID era

**DOI:** 10.1016/j.esmoop.2021.100055

**Published:** 2021-02-12

**Authors:** A. Toss, C. Isca, M. Venturelli, C. Nasso, G. Ficarra, V. Bellelli, C. Armocida, E. Barbieri, L. Cortesi, L. Moscetti, F. Piacentini, C. Omarini, A. Andreotti, A. Gambini, R. Battista, M. Dominici, G. Tazzioli

**Affiliations:** 1 Department of Oncology and Hematology, Azienda Ospedaliero-Universitaria di Modena, Modena, Italy; 2 Department of Surgery, Medicine, Dentistry and Morphological Sciences with Transplant Surgery, Oncology and Regenerative Medicine Relevance, University of Modena and Reggio Emilia, Modena, Italy; 3 Pathology Unit, University Hospital of Modena, Modena, Italy; 4 Breast Cancer Screening Service, AUSL Modena, Modena, Italy; 5 Department of Medical and Surgical Sciences for Children and Adults, University of Modena and Reggio Emilia, Modena, Italy; 6 Unit of Breast Surgical Oncology, Azienda Ospedaliero-Universitaria di Modena, Modena, Italy; 7 Department of Diagnostic Imaging, Azienda Ospedaliero-Universitaria di Modena, Modena, Italy

**Keywords:** COVID-19, breast cancer, stage at diagnosis, mammographic screening

## Abstract

**Introduction:**

The present analysis aims to evaluate the consequences of a 2-month interruption of mammographic screening on breast cancer (BC) stage at diagnosis and upfront treatments in a region of Northern Italy highly affected by the severe acute respiratory syndrome-related coronavirus-2 (SARS-CoV-2) virus.

**Methods:**

This retrospective single-institution analysis compared the clinical pathological characteristics of BC diagnosed between May 2020 and July 2020, after a 2-month screening interruption, with BC diagnosed in the same trimester of 2019 when mammographic screening was regularly carried out.

**Results:**

The 2-month stop in mammographic screening produced a significant decrease in *in situ* BC diagnosis (−10.4%) and an increase in node-positive (+11.2%) and stage III BC (+10.3%). A major impact was on the subgroup of patients with BC at high proliferation rates. Among these, the rate of node-positive BC increased by 18.5% and stage III by 11.4%. In the subgroup of patients with low proliferation rates, a 9.3% increase in stage III tumors was observed, although node-positive tumors remained stable. Despite screening interruption, procedures to establish a definitive diagnosis and treatment start were subsequently carried out without delay.

**Conclusion:**

Our data showed an increase in node-positive and stage III BC after a 2-month stop in BC screening. These findings support recommendations for a quick restoration of BC screening at full capacity, with adequate prioritization strategies to mitigate harm and meet infection prevention requirements.

## Highlights

• Due to the COVID19 pandemic, several mammographic screening services were disrupted.
• A 2-month stop in BC screening led to decreased *in situ* BC and increased node-positive and stage III BC diagnosis.
• A major impact was on the subgroup of patients with BC at high proliferation rates.
• Despite screening interruption, procedures to start treatments were subsequently carried out without delay.
• Restoration of BC screening at full capacity with infection prevention requirements is recommended.

## Introduction

The coronavirus disease 2019 (COVID-19) pandemic has raised unprecedented challenges for patients, clinicians and health care systems. Clinicians have responded to the pandemic by trying to reorganize and adapt the allocation of health care resources, staff and infrastructure, in order to minimize exposure risks without compromising patient outcomes, especially in oncology settings.^1^ On these grounds, the American Society of Breast Surgeons (ASBrS), the National Accreditation Program for Breast Centers (NAPBC), the National Comprehensive Care Network (NCCN), the Commission on Cancer (CoC), the American College of Radiology (ACR) as well as the Italian Association of Medical Oncology (AIOM) provided preliminary guidance on the prioritization and treatment of breast cancer (BC) during this particular period.^2^^,^^3^ In parallel, many national and international associations, cancer centers and research groups published their recommendations, driven by the common goal to ring-fence hospital resources for COVID-19 patients by reorganizing BC management strategies.^4^, ^5^, ^6^, ^7^, ^8^, ^9^, ^10^ The majority of these guidelines agreed that population mammographic screening and screening of mutation carriers should be suspended until the pandemic subsides.

Recently, the Italian College of Breast Radiologists, an offshoot of the Italian Society of Medical Radiology (SIRM), provided recommendations for procedural prioritization of breast imaging and cancer diagnosis during the COVID-19 pandemic. The recommendations were mainly aimed at asymptomatic women falling into two categories: those who did not respond to the invitation for screening mammography after the onset of the pandemic and those who were informed of the suspension of the screening activity. These were recommended to postpone the check preferably within 3 months of the date of the check as originally scheduled, as long as the operating conditions allowed for it.^11^ Such recommendations were driven by the common goal to preserve hospital resources for COVID-19 patients, by deferring breast imaging procedures without compromising long-term outcomes for individual patients. Nevertheless, the real impact of a temporary mammographic screening suspension on BC outcomes remains uncertain.

Our retrospective single-institution analysis aimed to evaluate the consequences on BC diagnosis of a 2-month interruption of mammographic screening in a region of Northern Italy highly affected by the severe acute respiratory syndrome-related coronavirus-2 (SARS-CoV-2) virus.

## Materials and methods

This is a retrospective single-institution analysis carried out at Modena University Hospital in Emilia Romagna, a region of Northern Italy highly affected by the SARS-CoV-2 virus (123 000 cases as of 1 December 2020). In Emilia Romagna, the mammographic screening provided by the National Healthcare System offers an annual mammogram to all asymptomatic women aged 45-49 years and a biennial mammogram between 50 and 74 years. In the province of Modena, 83 078 women were invited in 2019, with an adhesion rate of 78.1% and cancer detection rates of 12.96/1000 and 6.19/1000 at first examination and at recalls, respectively. Due to the rapid spread of the COVID-19 pandemic from the end of February 2020, mammographic screening services were temporarily interrupted from 8 March 2020. On 15 May 2020, the service resumed but for two more months it operated at reduced capacity. As a result, only one-third of the expected women were allowed to visit, and those delayed over the previous months were fast-tracked. To date, the service is still working in pursuit of the twofold aim of avoiding large gatherings and sanitizing the equipment after each visit. Only two-thirds of the previously expected women could therefore visit. For the whole period, radiology services across the province have been available for the evaluation of symptomatic patients, screening of high-risk women (predisposing gene mutation carriers) and BC patient follow-up.

In the present analysis, we identified the clinical pathological characteristics of women diagnosed with BC in the province of Modena between May 2020 and July 2020, after screening interruption. We then compared them with patients diagnosed in the same trimester of 2019, when mammographic screening was carried out regularly. In particular, age at diagnosis, menopausal status, type of diagnosis, estrogen receptor (ER) and progesterone receptor (PR) status, MIB1, human epidermal growth factor receptor 2 (HER2) status, clinical stage, time from the first cytological or histological diagnosis to first surgical and/or oncological visit, and time from first surgical and/or oncological visit to surgery or neoadjuvant treatment start were evaluated. ER, PR and HER2 expression was determined according to the national pathology guidelines, which closely adhere to international standards.^12^ cTNM (clinical tumor–nodes–metastases) and clinical stages were evaluated according to the eighth edition of the American Joint Committee on Cancer (AJCC) Cancer Staging Manual.^13^

Standard descriptive analyses were carried out for clinical endpoints. For crude association analysis, categorical data were analyzed using Fisher's exact test (two-sided). Two-tailed *P* values below 0.05 were considered statistically significant. Statistical analyses were carried out using MedCalc Statistical Software version 14.8.1 (MedCalc Software, Ostend, Belgium).

## Results

### Clinical pathological characteristics of overall population

The clinical pathological characteristics of the patients included in the analyses are listed in Table 1. Between May 2019 and July 2019, 15 942 mammograms were carried out and 223 individuals were diagnosed with BC (221 women and 2 men). In the same trimester of 2020, 9052 mammograms were carried out and 177 patients were diagnosed (174 women and 3 men). No statistically significant difference in the distribution of menopausal status was observed between the two periods (*P* = 0.41). In 2020, screen-detected tumors decreased (though not significantly) from 136 (61%) to 94 (53.1%) (*P* = 0.127), whereas patients diagnosed through mammographic follow-up significantly increased from 7 (3.1%) to 25 (14.1%) (*P* = 0.0001). With regard to BC biological profile, 183 (85.1%) tumors in 2019 and 142 (83.1%) in 2020 showed positive ER status, 138 (75.8%) and 122 (74.9%) positive PR status, 70 (38.5%) and 70 (42.9%) presented MIB1 ≥ 20%, while 34 (18.7%) and 27 (16.6%) tumors exhibited overexpressed HER2. No statistically significant difference in the distribution of biological features was observed between the two periods (*P* = 0.89, *P* = 1, *P* = 0.44, *P* = 0.67).

**Table 1:** Clinical pathological characteristics of patients in the overall population according to year of diagnosis

	2019 (223 patients)		2020 (177 patients)		*P* value
	*N*	%	*N*	%	
Menopausal status					
Premenopausal	46	20.8	33	19	0.41^a^
Perimenopausal	16	7.2	8	4.6	
Postmenopausal	159	71.9	130	74.7	
Male	2		3		–
Unknown	0		3		–
Type of diagnosis					
Screen-detected	136	61	94	53.1	0.127
Symptomatic (self-reported)	60	26.9	41	23.2	0.418
Occasional radiological examination	20	9	17	9.6	0.863
Follow up for previous breast cancer	7	3.1	25	14.1	0.0001
Estrogen receptor					
≥10%	178	82.8	140	81.9	0.89^b^
1-9%	5	2.3	2	1.2	
0	32	14.9	29	16.9	
Unknown	8		6		–
Progesterone receptor					
≥10%	121	66.5	109	66.9	1^b^
1%-9%	17	9.3	13	8	
0	44	24.2	41	25.1	
Unknown	41		14		–
MIB1					
<20%	112	61.5	93	57	0.44
≥20%	70	38.5	70	42.9	
Unknown	41		14		–
HER2 status					
Negative	146	80.2	136	83.4	0.67
Positive	34	18.7	27	16.6	
2+	2	1.1	0	0	–
Unknown	41		14		–
Clinical T					
cTis	38	17	12	6.8	0.0021
cT1a	8	3.6	5	2.8	0.3115
cT1b	40	17.9	32	18.1	
cT1c	51	22.8	52	29.4	
cT2	72	32.2	51	28.8	0.445
cT3	7	3.1	10	5.6	0.2
cT4	4	1.8	14	7.9	0.006
cTx	4	1.8	1	0.5	–
Clinical N					
cN+	28	12.5	42	23.7	0.0034
cN0	193	86.5	131	74	
cNx	2	0.9	4	2.2	–
Clinical stage					
0 (*in situ*)	38	17.2	12	6.8	0.0021
IA	83	37.5	83	47.1	0.06
IIA	69	31.2	34	19.3	0.008
IIB	16	7.2	17	9.7	0.4
III	5	2.2	22	12.5	0.0001
IV	10	4.5	8	4.5	1
Unknown	2		1		–

Clinical N, clinical nodes; Clinical T, clinical tumor; HER2, human epidermal growth factor receptor 2.

a Premenopausal status + perimenopausal status versus postmenopausal status.

b Estrogen and progesterone receptor 0%-9% versus ≥10%.

**Figure 1:**
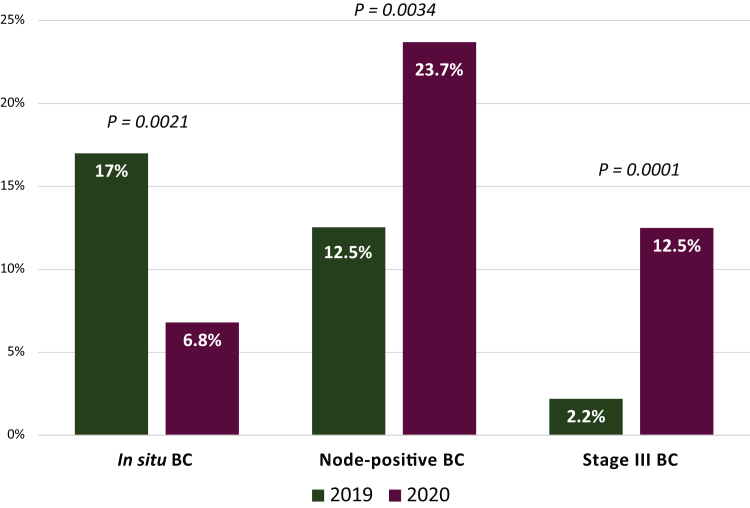
Comparison between rate of *in situ* BC, node-positive BC and stage III BC diagnosed in 2019 and 2020. BC, breast cancer.

A statistically significant decrease in *in situ* diagnosis was observed in 2020 (6.8% of BC diagnosis versus 17%; *P* = 0.0021) (Figure 1). Moreover, the rate of cT1 (89 patients, 50.3%), cT2 (51 patients, 28.8%) and cT3 (10 patients, 5.6%) tumors diagnosed in May-July 2020 did not significantly differ from the 2019 tumors (*P* = 0.3115, *P* = 0.445, *P* = 0.2, respectively). By contrast, cT4 tumors significantly increased from 4 (1.8%) in 2019 to 14 (7.9%) in 2020 (*P* = 0.006). Furthermore, the number of BCs with metastatic lymph nodes (cN+) at diagnosis significantly increased from 28 (12.5%) in 2019 to 42 (23.7%) in 2020 (*P* = 0.0034) (Figure 1). Accordingly, stage 0 (*in situ*) BCs significantly decreased from 38 (17.2%) to 12 (6.8%) (*P* = 0.0021), stage I BC decreased not significantly (83 patients, 37.5% versus 83 patients, 47.1%; *P* = 0.06), stage IIA BC significantly decreased (69 patients, 31.2% versus 34 patients, 19.3%; *P* = 0.008), stage IIB BC did not significantly vary (16 patients, 7.2% versus 17 patients, 9.7%; *P* = 0.4), stage III BC significantly increased (5 patients, 2.2% versus 22 patients, 12.5%; *P* = 0.0001) (Figure 1) and stage IV BC did not significantly vary (10 patients, 4.5% versus 8 patients, 4.5%; *P* = 1).

### Clinical pathological characteristics of patients diagnosed with invasive BC according to proliferation rate

Considering only those patients diagnosed with invasive BC at high proliferation rates (MIB1 ≥ 20%) (shown in Table 2), the distribution of cT1, cT2, cT3 and cT4 BC did not significantly change from 2019 to 2020. By contrast, the number of BCs with metastatic lymph nodes (cN+) at diagnosis remarkably increased from 20 (28.6%) to 33 (47.1%) (*P* = 0.0352). Overall, stage I, IIB and IV BC did not significantly vary (*P* = 0.1754, *P* = 0.65, *P* = 1), whereas stage IIA significantly halved from 35 cases (50%) in 2019 to 17 (24.3%) in 2020 (*P* = 0.0016) and stage III BC significantly more than tripled from 3 cases (4.3%) in 2019 to 11 (15.7%) in 2020 (*P* = 0.045).

**Table 2:** Clinical stage of patients diagnosed with invasive breast cancer according to proliferation rates (MIB1 ≥ 20% versus MIB1 < 20%) and years of diagnosis

	MIB1 ≥ 20%					MIB1 < 20%				
	2019 (70 patients)		2020 (70 patients)		*P* value	2019 (112 patients)		2020 (90 patients)		*P* value
	*N*	%	*N*	%		*N*	%	*N*	%	
Clinical T										
cT1a	1	1.4	0	0	1	7	6.2	5	5.5	0.8805
cT1b	8	11.4	5	7.1		31	27.7	24	26.7	
cT1c	15	21.4	19	27.1		35	31.2	32	35.6	
cT2	37	52.8	34	48.6	0.6	33	29.5	17	18.9	0.0742
cT3	4	5.7	5	7.1	1	3	2.7	5	5.5	0.4713
cT4	3	4.3	7	10	0.3	1	0.9	7	7.8	0.0238
cTx	2	2.8	0	0	–	2	1.8	0	0	–
Clinical N										
cN+	20	28.6	33	47.1	0.0352	8	7.1	7	7.8	1
cN0	48	68.6	36	51.4		104	92.8	81	90	
cNx	2	2.8	1	1.4	–	0	0	2	2.2	–
Clinical stage										
IA	14	20	22	31.4	0.1754	68	60.7	58	64.4	0.658
IIA	35	50	17	24.3	0.0016	32	28.6	17	18.9	0.137
IIB	11	15.7	14	20	0.65	5	4.5	3	3.3	0.734
III	3	4.3	11	15.7	0.045	2	1.8	10	11.1	0.0064
IV	6	8.6	6	8.6	1	4	3.6	1	1.1	0.384
Unknown	1	1.4	0	0	–	1	0.9	1	1.1	–

Clinical N, clinical nodes; Clinical T, clinical tumor.

By contrast, in patients diagnosed with invasive BC at low proliferation rate (MIB1 < 20%), only cT4 (1 patient, 1.8% in 2019 versus 7 patients, 7.8% in 2020; *P* = 0.0238) and stage III tumors (2 patients, 1.8% in 2019 versus 10 patients, 11.1% in 2020; *P* = 0.0064) were significantly increased, whereas node-positive BC remained stable (*P* = 1).

### Upfront treatments and median time to intervention

Overall, the median time from first cytological or histological diagnosis to first surgical and/or oncological visit was 19.9 days in 2019 (data available for 155 patients) and 18.3 days in 2020 (data available for 127 patients). Of all these patients, 32 (17.3%) underwent neoadjuvant treatment before surgery in 2019 (6 unknown) and 36 (28.1%) in 2020 (13 unknown) (*P* = 0.0793). For the 22 patients with available data in 2019, the median time from first surgical/oncological visit to neoadjuvant treatment start was 14.5 days, whereas for the 23 patients with available data in 2020, the median time from first surgical/oncological visit to neoadjuvant treatment start was 16.3 days. In 2019, by contrast, the median time from first surgical/oncological visit to breast surgery was 37.8 days (for 134 patients with available data), while the median time decreased to 30.9 days (for 127 patients) in 2020. Finally, of the 179 patients with known data undergoing upfront surgery in 2019, 142 (79.3%) underwent breast-conserving surgery, whereas of the 115 patients with known data in 2020, 83 (72.2%) underwent upfront breast-conserving surgery (*P* = 0.1618).

## Discussion

BC screening in the asymptomatic population leads to early diagnosis and treatment. This prospect results in improved survival and may avert BC deaths.^14^ Although asymptomatic women who have skipped their screening mammogram because of COVID-19 are recommended to reschedule the check preferably within 3 months,^11^ the real impact of temporary mammographic screening suspension on BC outcomes remains uncertain. Yong et al.^15^ have recently estimated the long-term clinical impact of BC screening interruptions in Canada using a validated mathematical model. The authors found that a 3-month interruption in BC screening could increase cases diagnosed at advanced stages and cancer deaths in 2020-2029. Moreover, longer interruptions and reduced volumes when screening resumes would further increase excess cancer deaths. Similarly, Sharpless^16^ reported the results of a comparable analysis in the USA, using the CISNET cancer simulation model. This analysis predicted approximately 5300 additional BC deaths in the USA over the next decade.

Due to the rapid spread of the COVID-19 pandemic in our region, the mammographic screening service of our province temporarily interrupted activities for about 2 months, followed by resumption to a reduced volume that still persists. This intervention, dictated by the need to contain the spread of the SARS-CoV-2 virus, produced a significant decrease in *in situ* BC diagnosis (−10.4%) and an increase in node-positive (+11.2%) and stage III BC (+10.3%). No significant differences in menopausal status and biological features (ER, PR, MIB1 and HER2 status) were observed between May 2020 and July 2020 and the same trimester in 2019. However, screen-detected tumors decreased by 7.9% (although not significantly) and diagnosis during follow-up mammograms for a previous history of BC significantly increased by 11%. Although BC was detected at later stages, diagnoses in symptomatic (self-reported) BC remained stable in the two trimesters. The increase in diagnosis among patients with a previous history of BC might be related to the temporary interruption of follow-up visits in the Oncology Division between March 2020 and May 2020. Between the end of May 2020 and the end of July 2020, all the postponed oncological visits were recovered, likely leading to an increase in the overall number of follow-up visits and thus BC diagnosis in these patients.

Nevertheless, it is noteworthy that screen-detected BC shows some peculiarities. In particular, women diagnosed through the mammographic screening programs usually present with tumors with luminal-like subtype, more frequently of low grade, small size and node-negative,^17^, ^18^, ^19^ and the significant decrease in *in situ* BC in our study population confirms these data. Some authors therefore believe that a delay of a few months in these diagnoses should not significantly impact on patient outcomes. For these reasons, in the present analysis BCs at low and high proliferation rates were also assessed separately. Our results confirmed that a major impact of screening disruption occurred in the subgroup of patients with BC at high proliferation rates (MIB1 ≥ 20%). For these, the rate of node-positive BC increased by 18.5% and stage III by 11.4%. Likewise, a 9.3% increase in stage III tumors was observed in the subgroup of patients with low proliferation rates, although node-positive tumors remained stable.

Despite screening interruption, our analysis showed that the procedures to obtain a definitive diagnosis and start treatment were subsequently carried out without delay. The median time from first cytological or histological diagnosis to first surgical and/or oncological visit and the median time from first oncological visit to neoadjuvant treatment start remained substantially unchanged. By contrast, the median time from first surgical visit to surgery even shrank by 7 days, due to the lower rate of *in situ* tumors and overall diagnosed BC as well as the reorganization of operating theaters in a dedicated COVID-free hospital. Finally, although the rate of neoadjuvant treatments increased by 10.8% and breast-conserving surgery decreased by 7.1%, the difference between the two periods was not statistically significant.

These results confirm the estimates obtained through the mathematical models of Yong et al.^15^ and Sharpless^16^ in terms of increased advanced stages at diagnosis. Additionally, since treatment of more advanced cancers generally involves more widespread use of systemic therapy and invasive surgery, we may also conclude that these delays in cancer diagnosis could be associated with increased morbidity and higher costs for our national health system. Nevertheless, a longer follow-up will be necessary to evaluate whether this delay will also have consequences on BC outcomes (mostly disease-free survival and overall survival). Furthermore, it is likely that the delay in BC diagnosis in our population depended both on the interruption of screening and on the subsequent (still ongoing) reduced volume of mammograms carried out. Therefore, the analysis of following trimesters will be necessary to properly estimate the real impact of these interruptions on the delay in diagnosis and rate of advanced-stage BC.

## Conclusions

Postponing screening procedures as a result of the COVID-19 pandemic was prudent and appropriate at one time. However, the spread, duration and future peaks of COVID-19 are unpredictable, and overlooking other life-threatening conditions such as BC for too long may turn one public health crisis into another. Our data showed an increase in node-positive and stage III BC after a 2-month stop in BC screening with resumption to a reduced volume. These findings support recommendations for the immediate and quick restoration of BC screening at full capacity, with adequate prioritization strategies to mitigate harm and comply with infection prevention requirements.
